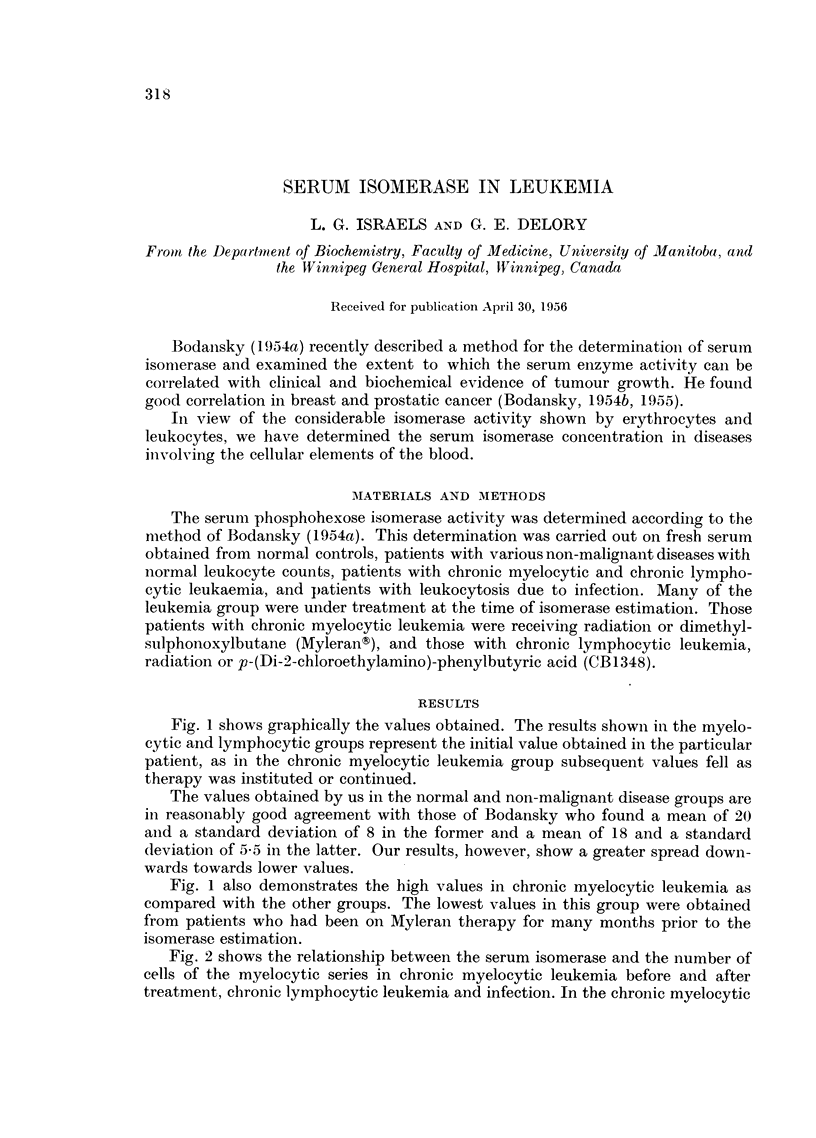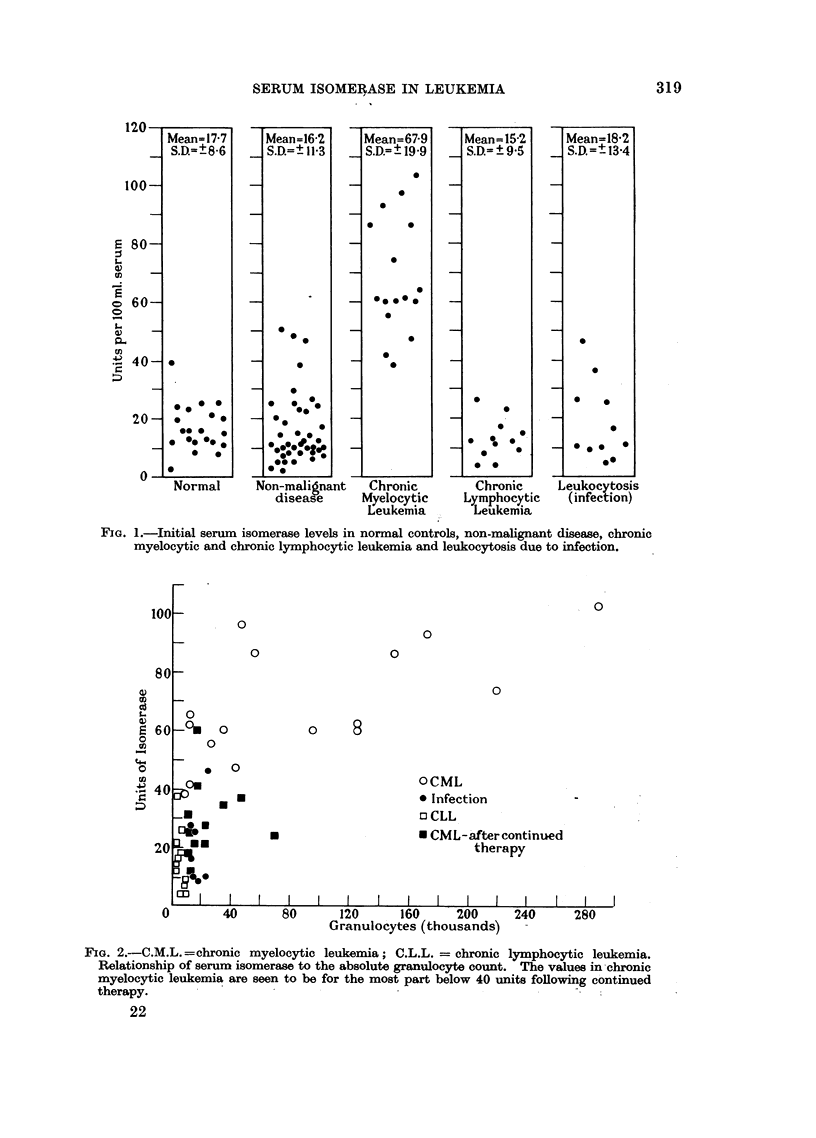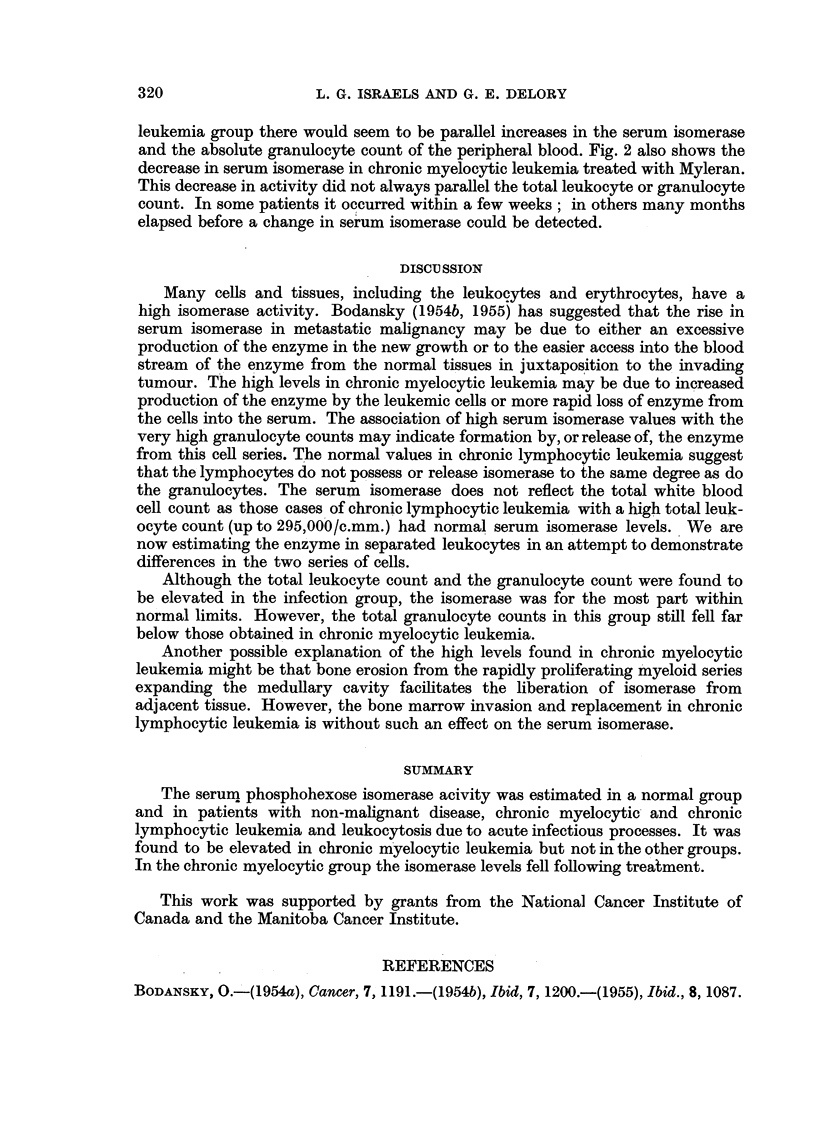# Serum Isomerase in Leukemia

**DOI:** 10.1038/bjc.1956.37

**Published:** 1956-06

**Authors:** L. G. Israels, G. E. Delory


### SERUM ISOMERASE IN LEUKEMIA

L. G. ISRAELS AND G. E. DELORY

*From the Department of Biochemistry, Faculty of Medicine, University of Manitoba, and the Winnipeg General Hospital, Winnipeg, Canada*

Received for publication April 30, 1956

Bodansky (1954*a*) recently described a method for the determination of serum isomerase and examined the extent to which the serum enzyme activity can be correlated with clinical and biochemical evidence of tumour growth. He found good correlation in breast and prostatic cancer (Bodansky, 1954*b*, 1955).

In view of the considerable isomerase activity shown by erythrocytes and leukocytes, we have determined the serum isomerase concentration in diseases involving the cellular elements of the blood.

### MATERIALS AND METHODS

The serum phosphohexose isomerase activity was determined according to the method of Bodansky (1954*a*). This determination was carried out on fresh serum obtained from normal controls, patients with various non-malignant diseases with normal leukocyte counts, patients with chronic myelocytic and chronic lymphocytic leukaemia, and patients with leukocytosis due to infection. Many of the leukemia group were under treatment at the time of isomerase estimation. Those patients with chronic myelocytic leukemia were receiving radiation or dimethylsulphonoxylbutane (Myleran®), and those with chronic lymphocytic leukemia, radiation or *p*-(Di-2-chloroethylamino)-phenylbutyric acid (CB1348).

### RESULTS

Fig. 1 shows graphically the values obtained. The results shown in the myelocytic and lymphocytic groups represent the initial value obtained in the particular patient, as in the chronic myelocytic leukemia group subsequent values fell as therapy was instituted or continued.

The values obtained by us in the normal and non-malignant disease groups are in reasonably good agreement with those of Bodansky who found a mean of 20 and a standard deviation of 8 in the former and a mean of 18 and a standard deviation of 5·5 in the latter. Our results, however, show a greater spread downwards towards lower values.

Fig. 1 also demonstrates the high values in chronic myelocytic leukemia as compared with the other groups. The lowest values in this group were obtained from patients who had been on Myleran therapy for many months prior to the isomerase estimation.

Fig. 2 shows the relationship between the serum isomerase and the number of cells of the myelocytic series in chronic myelocytic leukemia before and after treatment, chronic lymphocytic leukemia and infection. In the chronic myelocytic

Fig. 1.—Initial serum isomerase levels in normal controls, non-malignant disease, chronic myelocytic and chronic lymphocytic leukemia and leukocytosis due to infection.

Fig. 2.—C.M.L. = chronic myelocytic leukemia; C.L.L. = chronic lymphocytic leukemia. Relationship of serum isomerase to the absolute granulocyte count. The values in chronic myelocytic leukemia are seen to be for the most part below 40 units following continued therapy.

leukemia group there would seem to be parallel increases in the serum isomerase and the absolute granulocyte count of the peripheral blood. Fig. 2 also shows the decrease in serum isomerase in chronic myelocytic leukemia treated with Myleran. This decrease in activity did not always parallel the total leukocyte or granulocyte count. In some patients it occurred within a few weeks ; in others many months elapsed before a change in serum isomerase could be detected.

### DISCUSSION

Many cells and tissues, including the leukocytes and erythrocytes, have a high isomerase activity. Bodansky (1954*b*, 1955) has suggested that the rise in serum isomerase in metastatic malignancy may be due to either an excessive production of the enzyme in the new growth or to the easier access into the blood stream of the enzyme from the normal tissues in juxtaposition to the invading tumour. The high levels in chronic myelocytic leukemia may be due to increased production of the enzyme by the leukemic cells or more rapid loss of enzyme from the cells into the serum. The association of high serum isomerase values with the very high granulocyte counts may indicate formation by, or release of, the enzyme from this cell series. The normal values in chronic lymphocytic leukemia suggest that the lymphocytes do not possess or release isomerase to the same degree as do the granulocytes. The serum isomerase does not reflect the total white blood cell count as those cases of chronic lymphocytic leukemia with a high total leukocyte count (up to 295,000/c.mm.) had normal serum isomerase levels. We are now estimating the enzyme in separated leukocytes in an attempt to demonstrate differences in the two series of cells.

Although the total leukocyte count and the granulocyte count were found to be elevated in the infection group, the isomerase was for the most part within normal limits. However, the total granulocyte counts in this group still fell far below those obtained in chronic myelocytic leukemia.

Another possible explanation of the high levels found in chronic myelocytic leukemia might be that bone erosion from the rapidly proliferating myeloid series expanding the medullary cavity facilitates the liberation of isomerase from adjacent tissue. However, the bone marrow invasion and replacement in chronic lymphocytic leukemia is without such an effect on the serum isomerase.

### SUMMARY

The serum phosphohexose isomerase acivity was estimated in a normal group and in patients with non-malignant disease, chronic myelocytic and chronic lymphocytic leukemia and leukocytosis due to acute infectious processes. It was found to be elevated in chronic myelocytic leukemia but not in the other groups. In the chronic myelocytic group the isomerase levels fell following treatment.

This work was supported by grants from the National Cancer Institute of Canada and the Manitoba Cancer Institute.

### REFERENCES

Bodansky, O.—(1954*a*), *Cancer*, **7**, 1191.—(1954*b*), *Ibid*, **7**, 1200.—(1955), *Ibid.*, **8**, 1087.